# Bundle management strategy in reducing hospital-acquired pneumonia in hospitalized patients with mental disorders

**DOI:** 10.3389/fpsyt.2023.1184999

**Published:** 2023-06-02

**Authors:** Jingjing Han, Dan Li, Yan Rao, Gaohua Wang

**Affiliations:** ^1^Department of Infection Control, Renmin Hospital of Wuhan University, Wuhan, Hubei, China; ^2^Department of Pharmacy, Renmin Hospital of Wuhan University, Wuhan, Hubei, China; ^3^Animal Biosafety Level III Laboratory at the Center for Animal Experiment, Wuhan University School of Medicine, Wuhan, Hubei, China; ^4^Insititute of Neuropsychiatry, Renmin Hospital of Wuhan University, Wuhan, Hubei, China; ^5^Department of Psychiatry, Renmin Hospital of Wuhan University, Wuhan, Hubei, China

**Keywords:** mental disorders, healthcare-associated pneumonia, infection control, inpatients, management strategy

## Abstract

**Introduction:**

The incidence of hospital-acquired pneumonia (HAP) is high in the medical setting for mental disorders. To date, effective measurements for preventing HAP in hospitalized mental disorder patients are unavailable.

**Methods:**

This study was conducted at the Large-Scale Mental Health Center of Renmin Hospital of Wuhan University (Wuhan, China) in two phases: baseline phase (January 2017–December 2019) and intervention phase (May 2020–April 2022). In the intervention phase, the HAP bundle management strategy was implemented in the Mental Health Center, and the data on HAP were collected continuously for analysis.

**Results:**

A total of 18,795 and 9,618 patients were included in the baseline and intervention phases, respectively. The age, gender, ward admitted to, type of mental disorder, and Charlson comorbidity index did not differ significantly. After intervention, the rate of HAP occurrence decreased from 0.95 to 0.52% (*P* < 0.001). Specifically, the HAP rate decreased from 1.70 to 0.95% (*P* = 0.007) in the closed ward and from 0.63 to 0.35% (*P* = 0.009) in the open ward. The HAP rate in the subgroups was higher in patients with schizophrenia spectrum disorders (1.66 *vs*. 0.74%) and organic mental disorders (4.92 *vs*. 1.41%), and in those ≥65 years old (2.82 *vs*. 1.11%) but decreased significantly after intervention (all *P* < 0.05).

**Conclusion:**

The implementation of the HAP bundle management strategy reduced the occurrence of HAP in hospitalized patients with mental disorders.

## Introduction

According to the statistics of the World Health Organization (WHO), >1 billion people are affected by mental disorders, deeming them a global public health issue that cannot be ignored and that has brought heavy healthcare and financial burden worldwide (1). China is leading with the burden of mental disorders (2); >1.6 million patients were hospitalized for mental disorders in China in 2015 (3).

Hospital-acquired pneumonia (HAP) is defined as pneumonia not incubating at the time of hospital admission and occurring ≥48 h after hospital admission (4, 5). Previous studies have shown that HAP incidence is high in Chinese patients with mental disorders (6, 7). For instance, a large-scale specialized hospital for mental disorders in Sichuan (China) reported that the HAP incidence was 7.8% in middle-aged and elderly patients (≥50 years old) with schizophrenia (6). A large-scale specialized hospital for mental disorders in Taiwan reported that the HAP incidence was 14.7/1000 person-years in patients with severe mental disorders (>80% had schizophrenia or schizoaffective disorders) (7).

In recent years, the incidence of ventilator-associated pneumonia (VAP) in medical settings has been decreasing, while that of non–ventilator-associated HAP is increasing (8) with an incidence of about 1% (5, 9). Moreover, the total number of patients with HAP is 2-fold higher than the number of patients with VAP (9, 10). Most HAP (70.8%) occurs in the wards rather than the intensive care unit (ICU) (10). The disease could prolong the hospital stay, increase the overall medical cost, and mortality rate (11); however, it is the most underestimated hospitalization-related safety and economic issue. HAP is mainly induced by the aspiration or inhalation of pathogenic microorganisms in the form of aerosols or hydrogel microparticles (4, 5). Compared to regular patients, the occurrence of HAP in mental disorder patients has several unique characteristics, such as dietary and behavioral changes, different degrees of social function impairment, longer hospital stay, dysphagia (12), somatic comorbidity (13, 14), and dry mouth, drooling, and sedation induced by antipsychotics. Schizophrenia is a chronic and disabling mental illness. The previous findings suggest that HAP is particularly prevalent in patients with schizophrenia (6, 7). Moreover, organic mental disorders are more likely to be associated with older age and physical comorbidities such as dementia and cerebrovascular disease, and this may lead to an elevated risk of pneumonia and death. In addition to individualized factors, the transmission of pathogens capable of inducing pneumonia in the hospital also causes HAP. Previously, mental health settings/units have reported various outbreaks of *Streptococcus pneumonia* (15) and influenza (15, 16), indicating that hospitalized mental disorder patients may have a high risk of respiratory diseases.

Several studies have investigated the measures of preventing and managing VAP (9, 10, 17), and a perfect prevention and management system has been established. However, scientific evidence on the prevention and management of HAP is still limited. The major predictive factors for HAP could vary in patients without mechanical ventilation (18, 19). Previous studies have shown that interventional measures, such as improving hand hygiene, early activities, recognizing and managing dysphagia, and preventing viral infection (20), could reduce HAP occurrence in patients. The commonly applied infection prevention measures, such as hand hygiene, quarantine precautions, personal protective devices (gloves and masks), and disinfection of equipment and the environment, effectively reduce the risk of pathogen transmission in hospitals (21). However, the measures for effectively preventing the occurrence of HAP in mental disorder patients have not yet been investigated. Another survey showed that staff and patients in mental health settings have low overall compliance to infection prevention measures (22), which are manifested as follows: (1) mental disorder patients may exhibit cognitive impairment and thus are incapable of abiding by the infection prevention measures; (2) the understanding and implementation of common infection prevention and control measures could be insufficient for medical staff (23); (3) the hospitalization environment could be poor (24); (4) some essential equipment, such as ethanol sanitizer, could not be used due to safety concerns (25); (5) mental health settings encourage social interactions, group activities, and freedom of actions (26), which could increase the opportunity of cross-infection. Therefore, implementing infection prevention measures in mental health settings/units (27) is rather complex and could be influenced by factors, such as patients, medical staff, and organization (22). The bundle management strategy is the gathering of a series of evidence-based treatments and nursing, which plays a major role in effectively reducing VAP (28). In light of the current status and challenges in HAP prevention and management in mental disorder patients, as well as previous findings (20, 21), the present study developed a HAP bundle management strategy with respect to the levels of patients, medical staff, and organization and explored the effects on HAP in mental disorder patients.

## Patients and methods

### Study setting and subjects

This study was performed in the Mental Health Center of Renmin Hospital of Wuhan University (Wuhan, China) between January 2017 and April 2022. Patients hospitalized between January and April 2020 were not included due to the coronavirus disease-2019 (COVID-19) epidemic. This hospital is a large-scale general teaching institution with a large-scale mental health center with the highest number of beds for psychiatric patients in general hospitals in China (350 beds including 225 beds in open wards and 125 in closed wards across six wards). COVID-19 patients were not admitted to the Mental Health Center during the study period or included in the study.

The inclusion criteria were as follows: 1) hospitalized patients with the primary diagnosis of mental disorders according to the criteria described in the International Classification of Diseases-10 (ICD-10, codes F00-F99) and 2) Patients were hospitalized for >48 h.

The exclusion criteria were as follows: (1) incomplete data for analysis; (2) patients who died or were spontaneously discharged within 48 h after hospitalization; and (3) underwent mechanical ventilation.

The clinical research ethics committee of Renmin Hospital of Wuhan University approved this study (WDRY2022-K163), and informed consent was waived by the Ethics Committee of the hospital.

### Study design and intervention

This 5-year study consisted of two phases: baseline phase (January 2017–December 2019) and intervention phase (May 2020–April 2022).

In the baseline phase, the target surveillance of HAP was not performed in the mental health center, and pneumonia symptoms were not monitored daily for all hospitalized patients. Only a few patients and some medical staff wore masks, and management of patients with pneumonia or immunodeficiency was insufficient. Compliance with implementation of environmental cleaning and disinfection work was poor, such as environment disinfection was performed only 1 time/day, or unperformed in some wards sometimes, especially the disinfection of public areas. Hand hygiene was poor, and the monitoring of antipsychotics and side effects was limited. In May 2020, with the cooperation of the leaders of the hospital and multiple departments, including infection control, medicine, nursing, cleaning and disinfection management, and the mental health center, the risk was assessed according to the characteristics of mental disorder patients. Subsequently, a literature search was conducted according to evidence-based medicine-related guidelines and studies (18–28), and finally, the sophisticated management strategy was developed as follows:

1) Performing target surveillance of HAP: suspicious symptoms of pneumonia, such as cough, expectoration, and fever, were monitored daily for all the hospitalized patients to identify pneumonia patients in time. HAP occurrence was summarized and analyzed every month.
2) Improving the management of pneumonia patients: disinfection, hand hygiene, and wearing masks, were essential for contact isolation and respiratory isolation. For patients with pathogens detected, especially the infection or colonization of several drug-resistant microorganisms such as *Methicillin-resistant Staphylococcus aureus, Extended-spectrum-*β*-lactamases-producing Escherichia coli* or *Klebsiella pneumoniae, Carbapenem-resistant Escherichia coli* or *Klebsiella pneumoniae, Pseudomonas aeruginosa* or *Acinetobacter baumannii*, and respiratory viruses such as influenza, infection prevention measures were challenging; hence, isolation management was strengthened.
3) Enhancing disinfection, hand hygiene, and reasonable use of personal protective equipment: environment disinfection was performed two times/day, and additional attention was paid to the disinfection of public areas, such as activity rooms and recreation rooms for patients, toilets, elevators, and shared equipment and devices. The windows were opened regularly two times/day for ventilation. Compliance with hand hygiene was improved based on the WHO hand hygiene indications of “five moments for hand hygiene” (29). The antiseptic foam (water not required) was carried by doctors and nurses during ward rounds and also hung on the nursing cart (under supervision). Medical staff wore medical surgical masks during work; also, patients were required to wear masks. Extra bed was avoided. Regular education was conducted in all patients, such as hand hygiene, prevention, and control knowledge of HAP.
4) Protecting susceptible population: severe mental disorder patients with immunodeficiency, such as granulocytopenia or granulocytosis, and substantial malnutrition, “protective isolation,” was prescribed and effectuated.
5) Enhancing the surveillance of antipsychotics uses and focusing on the underlying diseases: management and surveillance of antipsychotics, such as clozapine, were enhanced. For instance, antipsychotic concentrations were monitored, and therapeutic drug monitoring (TDM) was strengthened, especially when adjusting drug dosage or suspecting poisoning or insufficient dosage in patients. The drug side-effect scale was filled. Information, including sialorrhea, dysphagia, and bedrest time of patients, was collected in the ward rounds every day, and symptomatic treatments were performed promptly. The patients resting in bed or with low mobility were encouraged to conduct off-bed activities, and the doses of sedatives were adjusted in time. Then, underlying diseases, blood glucose, liver and renal functions, albumin, and body weight were monitored, and the corresponding treatments were strengthened.
6) Standard training for this sophisticated HAP bundle management strategy, which was designed for hospitalized patients with mental disorders, was conducted for patients, medical staff, and organization. Implementation of all measures was supervised, and feedback was acquired, which promoted the effective and accurate implementation of the measures. The overall compliance of patients, medical staff, and organization to infection prevention measures is important for effectively preventing the occurrence of HAP in mental disorder patients (21, 22).

### Definition of HAP

Hospital-acquired pneumonia was diagnosed according to the criteria issued by the Centers for Disease Control and Prevention (USA) based on the comprehensive analysis of clinical manifestations, imaging findings of the chest, and laboratory examination results (30).

### Definition of mental disorders

The diagnoses of the mental disorders were made by professional psychiatrists in accordance with the 10th revision of the International Classification of Mental Disorders (ICD-10, codes F00-F99). According to the incidence rate of HAP of different types of mental disorders (6, 7) and the sample size of different types of mental disorders in this study, we divide the types of mental disorders into four categories. Schizophrenia spectrum disorder referred to Schizophrenia, schizotypal and delusional disorders (ICD-10 categories F20–F29), Mood-affective disorder referred to ICD-10 categories F30–F39, Organic mental disorder referred to ICD-10 categories F00–F09, and other mental disorders referred to ICD-10 categories F10–F19, F40–F48, F50–F59, F60–F69, F70–F79, F80–F89, F90–F98, and F99.

### Data collection

Clinical information, body temperature, blood routine results, and imaging findings of the lungs were collected through the real-time nosocomial infection surveillance system. Daily monitoring of suspicious symptoms was strengthened for all the hospitalized patients, and target surveillance of HAP was performed. Charlson comorbidity index (CCI) was used to assess the severity of somatic comorbidities (31). Hospital stay refers to the length of hospital stay from the patient's admission to discharge. The length of time until HAP occurrence refers to the hospitalization time from the patient's admission to the onset of HAP. HAP rate refers to the number of HAP events per 100 admissions or the number of HAP events per 1,000 days. HAP was diagnosed by professionals in infection control and doctors in charge of the bed, ensuring data quality. According to The World Health Organization hand hygiene observation method (29), Compliance to hand hygiene (%) = actual times of executing hand hygiene in the observation time/anticipated times of executing hand hygiene × 100%. Correctness of hand hygiene (%) = times of correctly executing hand hygiene in the observation time/actual times of executing hand hygiene × 100%.

### Statistical analysis

The data were analyzed using SPSS 22.0 (IBM Corp., Armonk, NY, USA) and Prism 8 (GraphPad Software, San Diego, CA, USA). Measurement data were tested for normality using the Kolmogorov–Smirnov method (sample size ≥50) or Shapiro–Wilk method (sample size < 50). Normally distributed measurement data are presented as the mean ± standard deviation (SD) and compared between groups using the t-test for independent samples. Non-normally distributed measurement data are shown as median [interquartile range (IQR)] and compared between groups using the Mann–Whitney U-test. Count data are presented as frequency (percentage) and analyzed using the chi-squared test or Fisher's exact test. The incidence before and after the intervention was compared using the unadjusted incidence relative risk (RR) ratios, defined as the ratio of events for a defined period. A *P*-value of < 0.05 was considered statistically significant.

## Results

### General characteristics of included patients

The age, gender, ward admitted to, type of mental disorder, and CCI did not differ significantly between the patients in the baseline and intervention phases (Table 1).

**Table 1:** General characteristics of study subjects during the baseline and intervention phases.

**Characteristic**	**Total (*****n** =* **28,413)**			**Closed ward (*****n** =* **8,254)**			**Open ward (*****n** =* **20,159)**		
	**Baseline (*****n** =* **18,795)**	**Intervention (*****n** =* **9,618)**	***P*** **-value**	**Baseline (*****n** =* **5,519)**	**Intervention (*****n** =* **2,735)**	***P*** **-value**	**Baseline (*****n** =* **13,276)**	**Intervention (*****n** =* **6,883)**	***P*** **-value**
Gender, *n* (%)			0.091			0.425			0.214
Male	7,980(42.46%)	3,983(41.41%)		2,937(53.22%)	1,430(52.28%)		5,043(37.98%)	2,553(37.09%)	
Female	10,815(57.54%)	5,635(58.59%)		2,582(46.78%)	1,305(47.72%)		8,233(62.02%)	4,330(62.91%)	
Age (years), *n* (%)			0.956			0.054			0.242
≥65	886 (4.71%)	452 (4.70%)		191 (3.46%)	118 (4.31%)		695 (5.24%)	334 (4.85%)	
< 65	17,909(95.29%)	9,166(95.30%)		5,328(96.54%)	2,617(95.69%)		12,581(94.76%)	6,549(95.15%)	
Ward admitted to, *n* (%)			0.103						
Closed ward	5,519 (29.36%)	2,735(28.44%)							
Open ward	13,276(70.64%)	6,883(71.56%)							
Type of mental disorder, *n* (%)			0.697			0.686			0.785
Schizophrenia spectrum disorder^a^	3,735 (19.87%)	1,878(19.52%)		1,762(31.93%)	841 (30.75%)		1,973 (14.86%)	1,037(15.07%)	
Mood affective disorder^b^	10,828(57.61%)	5,565(57.86%)		2,817(51.04%)	1,411(51.59%)		8,011 (60.34%)	4,154(60.35%)	
Organic mental disorder^c^	590 (3.14%)	284 (2.95%)		211 (3.82%)	104 (3.80%)		379 (2.85%)	180 (2.62%)	
Others^d^	3,642 (19.38%)	1,891(19.66%)		729 (13.21%)	379 (13.86%)		2,913 (21.94%)	1,512(21.97%)	
Charlson comorbidity index, *n* (%)			0.925			0.164			0.243
0–1 points	16,557(88.09%)	8,477 (88.14%)		4,892(88.64%)	2,409(88.08%)		11,665(87.86%)	6,068(88.16%)	
2 points	1,130 (6.01%)	584 (6.07%)		283 (5.13%)	128 (4.68%)		847 (6.38%)	456 (6.62%)	
3 points and above	1,108 (5.90%)	557 (5.79%)		344 (6.23%)	198 (7.24%)		764 (5.75%)	359 (5.22%)	

^a^Schizophrenia spectrum disorder (ICD-10 categories F20–F29).

^b^Mood-affective disorder (ICD-10 categories F30–F39).

^c^Organic mental disorder (ICD-10 categories F00–F09).

^d^Others (ICD-10 categories F10–F19, F40–F48, F50–F59, F60–F69, F70–F79, F80–F89, F90–F98, F99).

### Occurrence rate of HAP in mental disorder patients in different wards

**Figure 1:**
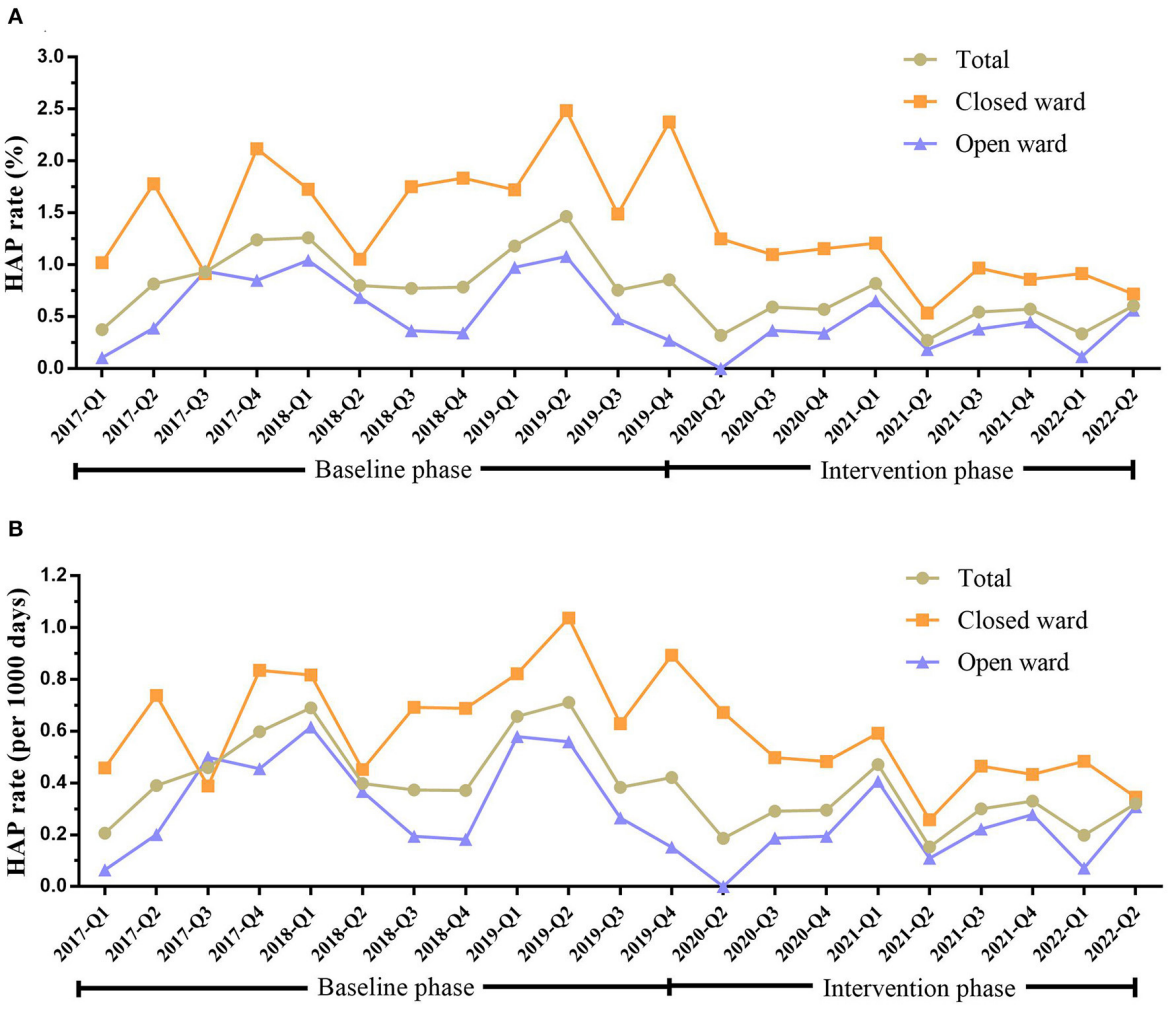
HAP rate in inpatients with mental disorders in total, closed ward, or open ward during the baseline and intervention phases. **(A)** HAP rate (%) in patients in total, closed ward, or open ward during the baseline and intervention phases. **(B)** The HAP rate (per 1,000 days) in patients in total, closed ward, or open ward during the baseline and intervention phases. HAP, hospital-acquired pneumonia; Q, quarter.

The rates of HAP occurrence in mental disorder patients in baseline and intervention phases are shown in Table 2 and Figure 1. HAP occurred in 178 patients in the baseline phase, of which 94 (52.81%) were in the closed ward. After intervention, the HAP rate reduced from 0.95 to 0.52% (*P* < 0.001). Specifically, the HAP rate reduced from 1.70 to 0.95% (*P* = 0.007) in the closed ward and reduced from 0.63 to 0.35% (*P* = 0.009) in the open ward. The hospital stay of patients was also significantly different between the baseline and intervention phases (*P* < 0.001).

**Table 2:** HAP rate and hospital stay in inpatients with mental disorders in total, closed ward, or open ward during the baseline and intervention phases.

**Characteristic**	**Total (*****n** =* **28,413)**				**Closed ward (*****n** =* **8,254)**				**Open ward (*****n** =* **20,159)**			
	**Baseline (*****n** =* **18,795)**	**Intervention (*****n** =* **9,618)**	**RR (95% CI)**	***P*** **-value**	**Baseline (*****n** =* **5,519)**	**Intervention (*****n** =* **2,735)**	**RR (95% CI)**	***P*** **-value**	**Baseline (*****n** =* **13,276)**	**Intervention (*****n** =* **6,883)**	**RR (95% CI)**	***P*** **-value**
No. of HAP patients	178	50			94	26			84	24		
No. of inpatients	18,795	9,618			5,519	2,735			13,276	6,883		
No. of patient-days	375,362	175,104			132,249	57,085			243,113	118,019		
HAP rate (%)	0.95%	0.52%	0.55 (0.40–0.75)	<0.001	1.70%	0.95%	0.55 (0.36–0.86)	0.007	0.63%	0.35%	0.55 (0.35–0.87)	0.009
HAP rate (per 1,000 days)	0.47‰	0.29‰	0.60 (0.44–0.82)	0.001	0.71‰	0.46‰	0.64 (0.42–0.99)	0.043	0.34‰	0.20‰	0.59 (0.37–0.93)	0.020
Hospital stay (days), median (IQR)	16.0 (11.0, 25.0)	16.0 (11.0, 22.0)		<0.001	20.0 (13.0, 30.0)	18.0 (13.0, 26.0)		<0.001	15.0 (10.0, 23.0)	15.0 (11.0, 21.0)		0.001
Length of time until HAP occurrence (days),^a^ median (IQR)	9.5 (6.0, 19.0)	11.0 (7.0, 20.0)		0.299	11.0 (8.0, 18.5)	13.0 (7.0, 25.0)		0.564	8.0 (5.5, 14.5)	10.0 (7.0, 17.5)		0.317

^a^Length of time from admission to HAP occurrence.

HAP, hospital-acquired pneumonia; RR, relative risk; 95% CI, 95% confidence interval; IQR, interquartile range.

### Rate of HAP occurrence in patients with different types of mental disorders

**Figure 2:**
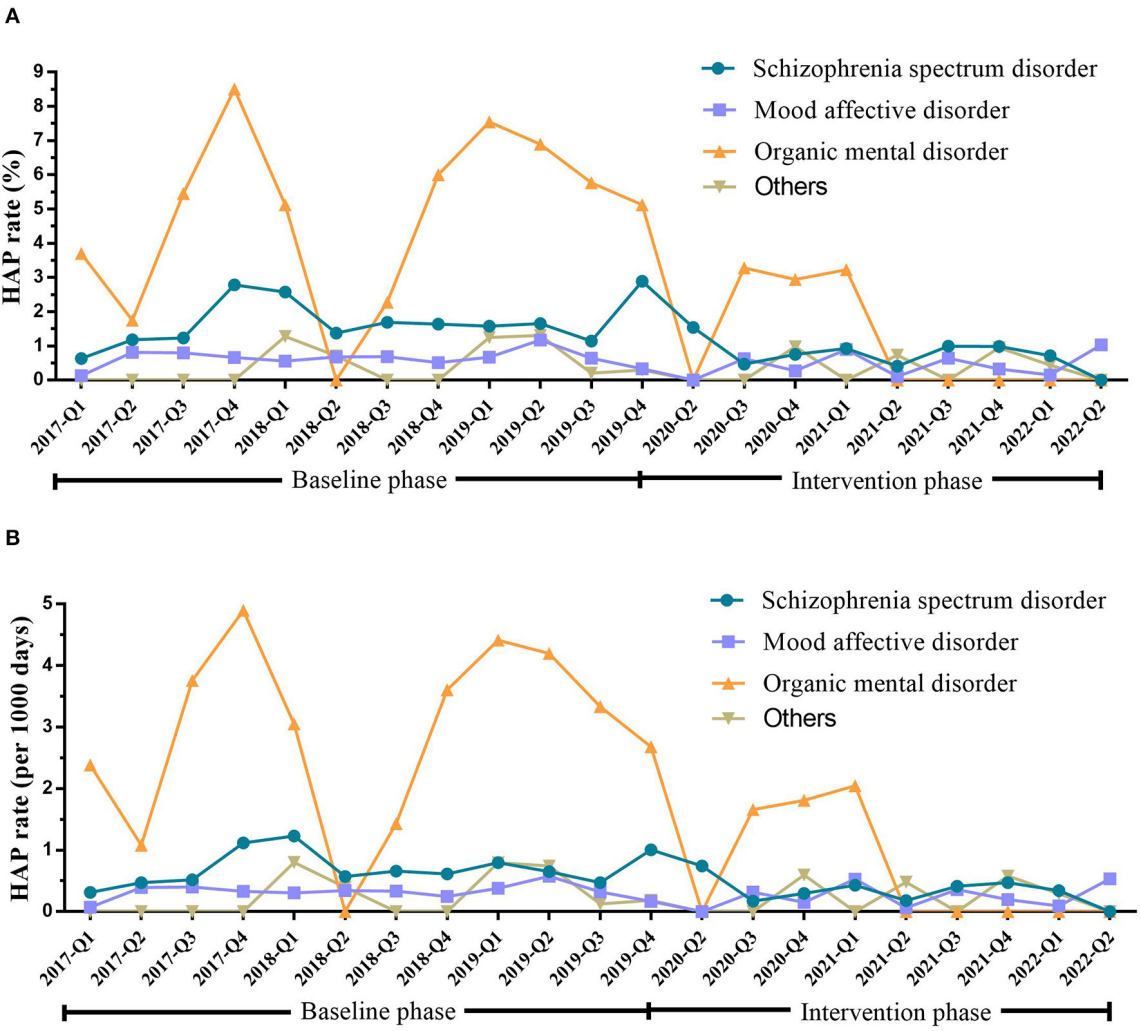
HAP rate in inpatients with different types of mental disorders during the baseline and intervention phases. **(A)** HAP rate (%) in patients with different types of mental disorders during the baseline and intervention phases. **(B)** HAP rate (per 1,000 days) in patients with different types of mental disorders during the baseline and intervention phases. Schizophrenia spectrum disorder (ICD-10 categories F20–F29; *n* = 5,613). Mood-affective disorder (ICD-10 categories F30–F39; *n* = 16,393). Organic mental disorders (ICD-10 categories F00–F09; *n* = 874). Others (ICD-10 categories F10–F19, F40–F48, F50–F59, F60–F69, F70–F79, F80–F89, F90–F98, F99; *n* = 5,533). HAP, hospital-acquired pneumonia; Q, quarter.

In the baseline phase, HAP occurred in 62 (34.83%), 72 (40.45%), 29 (16.29%), and 15 (8.43%) patients with schizophrenia spectrum, mood affective, organic mental, and other mental disorders, respectively. After intervention, the HAP rate reduced from 1.66 to 0.74% (*P* = 0.005) in patients with schizophrenia spectrum disorders and from 4.92 to 1.41% (*P* = 0.012) in the organic mental disorders (Table 3 and Figure 2).

**Table 3:** HAP rate and hospital stay in inpatients with different types of mental disorders during the baseline and intervention phases.

**Characteristic**	**Schizophrenia spectrum disorder^a^ (*n =* 5,613)**				**Mood affective disorder^b^ (*n =* 16,393)**				**Organic mental disorder^c^ (*n =* 874)**				**Others^d^ (*n* = 5,533)**			
	**Baseline (*****n** =* **3,735)**	**Intervention (*****n** =* **1,878)**	**RR (95% CI)**	***P*** **-value**	**Baseline (*****n** =* **10,828)**	**Intervention (*****n** =* **5,565)**	**RR (95% CI)**	***P*** **-value**	**Baseline (*****n** =* **590)**	**Intervention (*****n** =* **284)**	**RR (95% CI)**	***P*** **-value**	**Baseline (*****n** =* **3,642)**	**Intervention (*****n** =* **1,891)**	**RR (95% CI)**	***P*** **-value**
No. of HAP patients	62	14			72	24			29	4			15	8		
No. of inpatients	3,735	1,878			10,828	5,565			590	284			3,642	1,891		
No. of patient-days	89,799	42,834			213,412	97,814			9,764	4,498			62,387	29,958		
HAP rate (%)	1.66%	0.74%	0.44 (0.25–0.80)	0.005	0.66%	0.43%	0.65 (0.41–1.03)	0.063	4.92%	1.41%	0.28 (0.10–0.79)	0.012^e^	0.41%	0.42%	1.03 (0.44–2.43)	0.951
HAP rate (per 1000 days)	0.69‰	0.33‰	0.47(0.26–0.84)	0.010	0.34‰	0.24‰	0.73 (0.46–1.15)	0.175	2.97‰	0.89‰	0.30 (0.10–0.85)	0.016^e^	0.24‰	0.27‰	1.11 (0.47–2.62)	0.810
Hospital stay (days), median (IQR)	19.0 (12.0, 29.0)	19.0 (13.0, 28.0)		0.549	17.0 (11.0, 25.0)	15.0 (11.0, 22.0)		<0.001	14.0 (8.0, 20.0)	13.0 (10.0, 19.0)		0.464	15.0 (10.0, 21.0)	14.0 (10.0, 20.0)		0.117
Length of time until HAP occurrence (days),^f^ median (IQR)	13.5 (7.0, 23.0)	11.5 (5.0, 24.0)		0.737	10.0 (5.5, 20.0)	11.5 (7.0, 21.0)		0.244	7.0 (5.0, 11.0)	7.0 (5.5, 16.0)		0.852	7.0 (5.5, 9.5)	11.0 (7.5, 13.5)		0.040

^a^Schizophrenia spectrum disorder (ICD-10 categories F20–F29).

^b^Mood-affective disorder (ICD-10 categories F30–F39).

^c^Organic mental disorder (ICD-10 categories F00–F09).

^d^Others (ICD-10 categories F10–F19, F40–F48, F50–F59, F60–F69, F70–F79, F80–F89, F90–F98, F99).

^e^Fisher's exact probability method.

^f^Length of time from admission to HAP occurrence.

HAP, hospital-acquired pneumonia; RR, relative risk; 95% CI, 95% confidence interval; IQR, interquartile range.

### Rate of HAP occurrence in patients of different age groups

**Figure 3:**
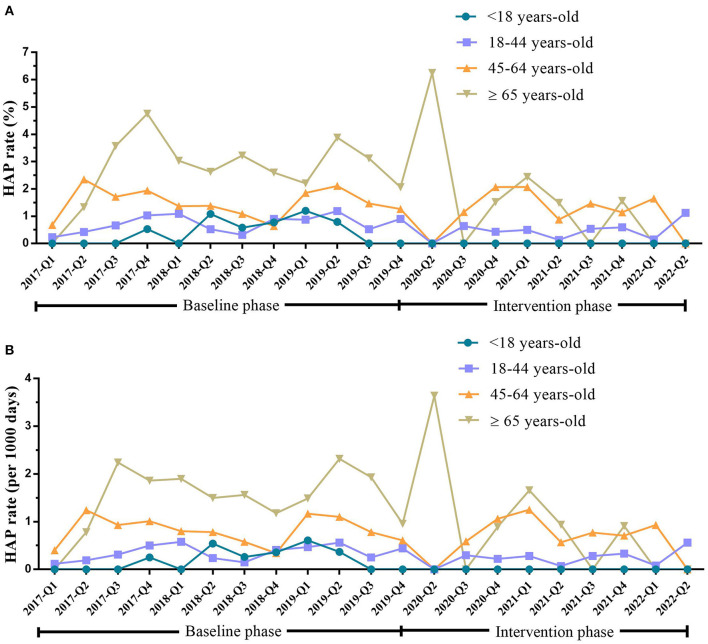
HAP rate in inpatients with mental disorders <18 years old, 18–44 years old, 45–64 years old, and ≥65 years old during the baseline and intervention phases. **(A)** HAP rate (%) in inpatients with mental disorders of <18 years old, 18–44 years old, 45–64 years old, and ≥65 years old during the baseline and intervention phases. **(B)** HAP rate (per 1,000 days) in inpatients with mental disorders of <18 years old, 18–44 years old, 45–64 years old, and ≥65 years old during the baseline and intervention phases. HAP, hospital-acquired pneumonia; Q, quarter.

In the baseline phase, HAP occurred in 10 (5.62%), 85 (47.75%), 58 (32.58%), and 25 (14.04%) patients aged < 18 years, 18–44 years, 45–64 years, and ≥65 years, respectively. After intervention, the HAP rate reduced from 0.42 to 0% (*P* = 0.002) in patients aged <18 years, from 0.73 to 0.44% (*P* = 0.030) in patients aged 18–44 years, and from 2.82 to 1.11% (*P* = 0.045) in patients aged ≥65 years (Table 4 and Figure 3).

**Table 4:** HAP rate and hospital stay in inpatients of different age brackets during the baseline and intervention phases.

**Characteristic**	**<18 years-old (*n =* 4,614)**				**18–44-years-old (*n =* 16,869)**				**45–64-years-old (*n =* 5,592)**				**≥65-years-old (*n =* 1,338)**			
	**Baseline (*****n** =* **2,370)**	**Intervention (*****n** =* **2,244)**	**RR (95% CI)**	***P*** **-value**	**Baseline (*****n** =* **11,659)**	**Intervention (*****n** =* **5,210)**	**RR (95% CI)**	***P*** **-value**	**Baseline (*****n** =* **3,880)**	**Intervention (*****n** =* **1,712)**	**RR (95% CI)**	***P*** **-value**	**Baseline (*****n** =* **886)**	**Intervention (*****n** =* **452)**	**RR (95% CI)**	***P*** **-value**
No. of HAP patients	10	0			85	23			58	22			25	5		
No. of inpatients	2,370	2,244			11,659	5,210			3,880	1,712			886	452		
No. of patient-days	48,102	39,327			240,135	98,169			71,020	30,163			16,105	7,445		
HAP rate (%)	0.42%	0 %	0.10 (0.01–0.82)	0.002^a^	0.73%	0.44%	0.60 (0.38–0.96)	0.030	1.49%	1.28%	0.86 (0.52–1.41)	0.543	2.82%	1.11%	0.38 (0.15–1.01)	0.045
HAP rate (per 1,000 days)	0.21‰	0‰	0.12 (0.02–0.96)	0.003^a^	0.35‰	0.23‰	0.66 (0.42–1.05)	0.077	0.82‰	0.73‰	0.89 (0.55–1.46)	0.651	1.55‰	0.67‰	0.43 (0.16–1.13)	0.078
Hospital stay (days), median (IQR)	17.0 (11.0, 26.0)	15.0 (11.0, 21.0)		<0.001	17.0 (11.0, 26.0)	16.0 (12.0, 23.0)		<0.001	15.0 (10.0, 22.0)	15.0 (11.0, 21.0)		0.986	14.0 (10.0, 20.0)	14.0 (10.0, 21.0)		0.754
Length of time until HAP occurrence (days),^b^ median (IQR)	16.7± 12.1	0.0		0.220	11.0 (6.0, 20.0)	11.0 (7.2, 20.0)		0.691	8.0 (5.2, 16.5)	9.0 (6.8, 14.5)		0.589	7.0 (5.0, 10.0)	22.0± 16.7		0.034

^a^Fisher's exact probability method.

^b^Length of time from admission to HAP occurrence.

HAP, hospital-acquired pneumonia; RR, relative risk; 95% CI, 95% confidence interval.

IQR, interquartile range.

### Implementation of HAP bundle management strategy

After the implementation of the HAP bundle management strategy, the compliance of medical staff with hand hygiene increased from 76.32% (58/76) to 93.29% (139/149) (*P* < 0.001), and the correct rate of hand hygiene in medical staff increased from 71.05% (54/76) to 89.93% (134/149) (*P* < 0.001). The consumption of antiseptic foam (water not required) increased from 11,850.00 mL/ward/year (0.37 mL/day/bed) to 45,833.33 mL/ward/year (1.57 mL/day/bed). The consumption of chlorine-containing disinfectants increased from 400 g active chlorine/ward/month to 600 g active chlorine/ward/month. The consumption of medical surgical masks increased from 250 masks/ward/month to 2,575 masks/ward/month.

## Discussion

To the best of our knowledge, this is the first study exploring the rate of HAP occurrence in mental disorder patients in a general hospital and the effectiveness of implementing the bundle management strategy in reducing HAP occurrence. The findings of this 5-year study showed that the implementation of the HAP bundle management strategy reduced the occurrence of HAP in mental disorder patients.

The findings of this study showed that in the baseline phase, the HAP rate was 0.95% (0.47/1,000 patient-days) in the hospitalized mental disorder patients, which was similar to the HAP rate in regular patients hospitalized in other general hospitals (approximately 1%) (5, 9); however, it was lower than the HAP rate in middle-aged or elderly patients with schizophrenia (6) or severe mental disorders (7) admitted in specialized hospitals for mental disorders. The HAP rate was higher in closed wards, in patients with schizophrenia spectrum disorders, in patients with organic mental disorders, and in patients aged ≥65 years than in regular patients in other general hospitals (5, 9). After the HAP bundle management strategy was implemented, the HAP rate in mental disorder patients reduced from 0.95 to 0.52%, and the hospital stay was reduced.

In this study, the high HAP rate in the closed ward could be associated with the characteristics of hospitalized patients. Patients hospitalized in the closed ward also exhibited severe mental disorders; the capability of self-perception and social functions was damaged to different degrees, and restraining the patients was difficult. Such patients had poor self-caring and self-controlling capabilities and poor personal hygiene. Therefore, closed wards require stringent environment disinfection, open window for ventilation, and hand hygiene. Patients with severe mental disorders might also have dysphagia. The treatment of severe mental disorders was complex, which increased the possibility of using drugs, such as clozapine, while long-term use of high-dose antipsychotics induces side effects and somatic diseases. The findings of this study showed that the HAP rate was high in patients with organic mental disorders or schizophrenia spectrum disorders. Schizophrenia is the most common chronic disabling mental disease (32). Previous studies have demonstrated a high incidence of pneumonia in schizophrenia patients (33). Patients with organic mental disorders could have an underlying “organic basis” that could lead to comorbid physical illnesses (34). For instance, several patients with advanced age, dementia, cerebrovascular disorders, and various comorbidities faced an increased risk of pneumonia and death.

According to features of mental disorders and the low compliance to infection-preventing measures in mental health settings/units (22), the present study developed and implemented the HAP bundle management strategy with respect to the levels of patients, medical staff, and organization and explored the effects on HAP in mental disorder patients. This HAP bundle management strategy focused on the characteristics of wards for mental disorders and features of diseases, ensuring the scientific relevance and feasibility of the management strategy. For instance, the strategy highlighted the importance of terminating the transmission in the hospital, such as enhancing the management of infected patients, cutting off the transmission route, and protecting the susceptible population as mental disorder patients might have cognitive impairment and are incapable of abiding by the measures of infection prevention. In order to consider patients' safety, the doctors and nurses carried antiseptic foam (water not required) during ward rounds and hung it on the nursing cart (under supervision). Regarding the poor environment in the psychiatric ward, training on air disinfection in the ward, material surface, public area, and public equipment, and management of disinfection frequency was enhanced. Regarding the encouragement of social interactions, and group activities for mental disorder patients (26), this study enhanced the disinfection in the activity rooms/recreation rooms for patients and prevented the gathering of pneumonia patients with other patients. Furthermore, this study enhanced the attention and management of antipsychotics and underlying diseases, monitored the antipsychotics concentrations, filled the “drug side-effect scale,” and focused on sialorrhea, dysphagia, and duration of bedrest. The cooperation and overall compliance of patients, medical staff, and organization to infection prevention measures is important for effectively preventing the occurrence of HAP in mental disorder patients (21, 22). An intensive focus by hospital leaders and mental health center staff guarantees the successful implementation of the bundle management strategy. The target surveillance of HAP promotes the implementation of preventive measures and ensures the standardization of outcome estimation.

## Limitations

First, the study quality could be improved further. This study was not a randomized controlled trial (RCT), and concurrent controls were not included; thus, the evidence-based evidence grade was low. Therefore, the present study could not provide definite conclusions on the effectiveness of specific interventional measures. Thus, we speculated that the HAP bundle management strategy reduces the occurrence of HAP in mental disorder patients. Second, this was a single-center study, and thus the results should be interpreted with caution. Third, the study was performed in the mental health center of a tertiary general hospital, and additional discussions are needed for the bundle management strategy of HAP related to other disorders or specialized hospitals. Finally, the sample size was large, and the severity of mental disorders was classified by manual scaling in the early phase of this study; thus, the severity of mental disorders of all patients could not be acquired on admission.

## Conclusion

This study underscored the importance of HAP surveillance in hospitalized mental disorder patients, while implementing the HAP bundle management strategy reduced HAP occurrence in mental disorder patients hospitalized in closed or open wards.

## Data availability statement

The original contributions presented in the study are included in the article/supplementary material, further inquiries can be directed to the corresponding authors.

## Ethics statement

The Clinical Research Ethics Committee of Renmin Hospital of Wuhan University approved this study (WDRY2022-K163). Written informed consent was waived by the Ethics Committee of the hospital.

## Author contributions

JH, YR, and GW conceived and designed the experiment and contributed to revise the manuscript. JH performed the research, collected the data, analyzed the results, wrote the manuscript, carried out literature search, and submitted the paper. JH and DL analyzed the data. All authors read and approved the final manuscript.
